# Supplementary material for: Heat adaptation of phage T7 under an extended genetic code
Source: Virus Evol. 2021 Dec 1;7(2):veab100. doi: 10.1093/ve/veab100 (PMC8923235; doi:10.1093/ve/veab100)
Supplement: veab100_Supp [file veab100_supp.zip › Sequencing_Analysis.zip!/Sequencing_Analysis/S1/evidence/SNP_10.html]

BRESEQ :: Evidence


| Predicted mutation | | | | | | |
| --- | --- | --- | --- | --- | --- | --- |
| evidence | seq id | position | mutation | annotation | gene | description |
| RA | NC\_001604.1 | 28,691 | A→C | intergenic (–/–) | – / – | –/– |

| Read alignment evidence... | | | | | | | | | | | |
| --- | --- | --- | --- | --- | --- | --- | --- | --- | --- | --- | --- |
|  | seq id | position | | ref | new | freq | score (cons/poly) | reads | annotation | genes | product |
| \* | NC\_001604.1 | 28,691 | 0 | A | C | 98.8% | 11478.5 / NA | 3815 | intergenic (–/–) | *–/–* | –/– |
| Reads supporting (aligned to +/- strand):  **ref** base A (17/28);  **new** base C (1898/1853);  **total** (1918/1897) | | | | | | | | | | | |
| Fisher's exact test for biased strand distribution *p*-value = 9.93e-02 | | | | | | | | | | | |
| Kolmogorov-Smirnov test that lower quality scores support variant *p*-value = 1.00e+00 | | | | | | | | | | | |

Only 100 of 3875 total aligned reads displayed.  

`AGGTATGGGCTATCGTGCAGCAACCACTCAGGCCGAACAGCCAAGGTCAAGCCTATTGGACACCATTGGTCGGTTCGCTAAGGCTGGTGCCGATATGTATACCGCTAAGGAACAACGAGCACGAGACCTAGCTGATGAACGCTCTAACGAGATTATCCGTAAGCTGACCCCTGAGCAACGTCGAGAAGCTCTCAACAACGGGACCCTTCTGTATCAGGATGACCCATACGCTATGGAAGCACTCCGAGTCAAGACTGGTCGTAACGCTGCGTATCTTGTGGACGATGACGTTATGCAGAAGATAAAAGAGGGTGTCTTCCGTACTCGCGAAGAGATGGAAGAGTATCGCCATAGTCGCCTTCAAGAGGGCGCTAAGGTATACGCTGAGCAGTTCGGCATCGACCCTGAGGACGTTGATTATCAGCGTGGTTTCAACGGGGACATTACCGAGCGTAACATCTCGCTGTATGGTGCGCATGATAACTTCTTGAGCCAGCAAGCTCAGAAGGGCGCTATCATGAACAGCCGAGTGGAACTCAACGGTGTCCTTCAAGACCCTGATATGCTGCGTCGTCCAGACTCTGCTGACTTCTTTGAGA  >  NC_001604.1/28393‑28991`  

                                                                                                                                                                                                                                                                                                          `|`                                                                                                                                                                                                                                                                                                              
`aagagacagCTATCGTGCAGCAACCACTCAGGCCGAACAGCCAAGGTCAAGCCTATTGGACACCATTGGTCGGTTCGCTAAGGCTGGTGCCGATATGTATACCGCTAAGGAACAACGAGCACGAGACCTAGCTGATGAACGCTCTAACGAGATTATCCGTAAGCTGACCCCTTAGCAACGTCGAGAAGCTCTCAACAACGGGACCCTTCTGTATCAGGATGACCCATACGCTATGGAAGCACTCCGAGTCAAGACTGGTCGTAACGCTGCGTATCTTGTGGACGATGACGTTATGcagcag                                                                                                                                                                                                                                                                                                            <  1:39280/293‑1 (MQ=255)`  
     `ggggCTATCGGGCAGCAACCACTCAGGCCGAACAGCCATGGTCAAGCCTATTGGACACCATTGGTCGGTTCGCTAAGGCTGGTGCCGATATGTATACCGCTAAGGAACAACGAGCACGAGACCTAGCTGATGAACGCTCTAACGAGATTATCCGTAAGCTGACCCCTGAGCAACGTCGAGAAGCTCTCAACAACGGGACCCTTCTGTATCAGGATGACCCATACGCTATGGAAGCACTCCGAGTCAAGACTGGTCGTAACTCTGCGTATCTTGTGGACGATGACGTTATGCAGCAGATaaa                                                                                                                                                                                                                                                                                                       <  1:375003/300‑1 (MQ=255)`  
      `gggCTATCGTGCAGCAACCACTCAGGCCGAACAGCCAAGGTCAAGCCTATTGGACACCATTGGTCGGTTCGCTAAGGCTGGTGCCGATATGTATACCGCTAAGGAACAACGAGCACGAGACCTAGCTGATGAACGCTCTAACGAGATTATCCGTAAGCTGACCCCTGAGCAACGTCGAGAAGCTCTCAACAACGGGACCCTTCTGTATCAGGATGACCCATACGCTATGGAAGCACTCCGAGTCAAGACTGGTCGTAACGCTGCGTATCTTGTGGACGATGACGTTATGCAGCAGATaaaa                                                                                                                                                                                                                                                                                                      >  1:512674/1‑301 (MQ=255)`  
       `tataagagacaggcaACCACTCAGGCCGAACAGCCAAGGTCAAGCCTATTGGACACCATTGGTCGGTTCGCTAAGGCTGGTGCCGATATGTATACCGCTAAGGAACAACGAGCACGAGACCTAGCTGATGAACGCTCTAACGAGATTATCCGTAAGCTGACCCCTGAGCAACGTCGAGAAGCTCTCAACAACGGGACCCTTCTGTATCAGGATGACCCATACGCTATGGAAGCATTCCGAGTCAAGACTGGTCGTAACGCTGCGTATCTTGTGGACGATGACGTTATGCAGCAGATAAAag                                                                                                                                                                                                                                                                                                     <  1:307329/289‑1 (MQ=255)`  
       `ggtaatagggaagcaACCATACATACCGTACAGACAAGGTGAAGCCTAGTGGAAACCATTGGTAGGGTCGCTAAGGCTGGTGCCGATATGTATAACGCGAAGGAACAACGAGCACGAGAGGTAGCTGATGAACGCTCTAACGAGATTATCAGTAAGCTGTCCCCAGAGCAACGTCGAGACGCTCGCAACAACGGAAGCCTTCTGTATAAGGATGACCCATACGCTATGGAAGCACTCCGAGTCAAGACTGGTCGTAACGCTGCGTATCTGGTGGAAGATGACGTTATGCAGCAGATAAAag                                                                                                                                                                                                                                                                                                     <  1:223713/290‑1 (MQ=255)`  
       `ggCTATCGTGCAGCAACCACTCAGGCCGAACAGCCAAGGTCAAGCCTATTGGACACCAGTGGTCGGTTCGCTAAGGCTGGTGCCGATATGTATACCGCTAAGGAACAACGAGCACGAGACCTAGCTGATGAACGCTCTAACGAGATTATCCGTAAGCTGACCCCTGAGCAACGTCGAGAAGCTCTCAACAACGGGACCCTTCTGTATCAGGATGACCCATACGCTATGGAAGCACTCCGAGTCAAGACTGGTCGTAACGCTGCGTATCTTGTGGACGATGACGTTATGCAGCAGATAAAag                                                                                                                                                                                                                                                                                                     <  1:544056/301‑1 (MQ=255)`  
       `ggCTATCGTGCAGCAACCAATCAGGCCGAAAAGCCAAGGTAAAGCCTATTGGAAACCATTGGTCGGTGCGCCAAGGCTGGTGCCGATATGTATACCGCTAAGGAACAACGAGCACGAGACCTAGCTGATGAACGCTCTAACGAGATTATCCGTAAGCTGACCCCTGAGCAACGTCGAGAAGCGCTCAACAACGGGACCCTTCTGTATCAGGATGACCCATACGCTATGGAAGCACTCCGAGTCAAGACTGGTCGTAACGCTGCGTATCTTGTGGACGATGACGTTATGCAGCAGATAAAag                                                                                                                                                                                                                                                                                                     <  1:430489/301‑1 (MQ=255)`  
         `cTATCGTGCAGCAAACGTTCAGGCCGAGCAGCCAAGGTCAAGCCTATTGGACACAATTGGTCGGTTCGCTAAGGCTGGTGCCGAAATGTATAACGCTAATGAACAACGAGCACGACACCTAGCTGATGAACGCTCTAACGAGATTATCCGTATGCGGACCCCTGAGCAACGTCGAGAAGCTCTCAACAACGGGACCCTTCTGTATCAGGATGACCCATACGCTATGGAAGCACTCCGAGTCAAGACTGGTCGTAACGCTGCGTATCTTGTGGACGATGACGTTATGCAGCAGATAAAagag                                                                                                                                                                                                                                                                                                   <  1:531861/301‑1 (MQ=255)`  
         `atATCGTGCAGCAACCACTAAGGCCGAACACCCAAGGACAAGCCTAGTGGACACCATTAGTCGGGTCGCTAAGGCTGGTGCCGATATGTATACCGCTAATGAACAACGAGCACGAGACCTAGCTGATGAACGCTCTAACGAGATTATCCGTAAGCTGACCCCTGAGCAACGTCGAGAAGCTCTCAACAACGGGACCCTTCTGTATCAGGATGACCCATACGCTATGGAAGCACTCCGAGTCAAGACCGGTCTTAACGCTGCGTATCTTGTGGACGATGACGTTATGCAGCAGATAAAagag                                                                                                                                                                                                                                                                                                   <  1:100156/300‑1 (MQ=255)`  
           `tataagagacagccACTCAGGCCGAACAGCCAAGGTCAAGCCTATTGGACACCATTGGTCGGTTCGCTAAGGCTGGTGCCGATATGTATACCGCTAAGGAACAACGAGCACGAGACCTAGCTGATGAACGCTCTAACGAGATTATCCGTAAGCTGACCCCTGAGCAACGTCGAGAAGCTCTCAACAACGGGACCCTTCTGTATCAGGATGACCCATACGCTATGGAAGCACTCCGAGTCAAGACTGGTCGGAACGCTGCGTATCTTGTGGACGATGACGTTATGCAGCAGATAAAAGAggg                                                                                                                                                                                                                                                                                                 <  1:398372/289‑1 (MQ=255)`  
           `aTCGTGCAGCAACCACTCAGGCCGAACAGCCAAGGTCAAGCCTATTGGACACCATTGGTCGGTTCGCTAAGGCTGGTGCCGATATGTATACCGCTAAGGAACAACGAGCACGAGACCTAGCTGATGAACGCTCTAACGAGATTATCCGTAAGCTGACCCCTGAGCAACGTCGAGAAGCTCTCAACAACGGGACCCTTCTGTATCAGGATGACCCATACGCTATGGAAGCACTCCGAGTCAAGACTGGTCGTAACGCTGCGTATCTTGTGGACGATGACGTTATGCAGCAGATAAAAGAggg                                                                                                                                                                                                                                                                                                 >  1:7031/1‑301 (MQ=255)`  
                    `caACCAAGCAGGCCGAACAGCCAAGGTCAAGCCTATTGGACACCATTGGTCGGTTCGCTAAGGCTGGTGCCGATATGTATACCGCTAAGGAACAACGAGCACGAGACCTAGCTGATGAACGCTCTAACGAGATTATCCGTAAGCTGACCCCTGAGCAACGTCGAGAAGCTCTCAACAACGGGACCCTTCTGTATCAGGATGACCCATACGCTATGGAAGCACTCCGAGTCAAGACTGGTCGTAACGCTGCGTATCTTGTGGACGATGACGTTATGCAGCAGATAAAAGAGGGTGTCTTCCg                                                                                                                                                                                                                                                                                        <  1:3404/301‑1 (MQ=255)`  
                        `cACTCAGACCGAACAGCCAAGGTCAAGCCTATTGGACACCATTGGTCGGTTCGCTAAGGCTGGTGCCGATATGTATACCGCTAAGGATCAACGAGCACTTGACCTAGCTGATGAACGCTCTAACGAGATTATCCGTAAGCTGACCCCTGCGCAACGTCTAGAAGCTCTCAACAACGGGACCCTTCTGTATCAGCATGACCCATACGCCATGGAAGCACTCCGAGTCAAGACTGGTCGTAACGCTGCGTATCTTGTGGACGATGACGTTATGCAGCCGATAAAcgactgctgtctcatatac                                                                                                                                                                                                                                                                                    >  1:87207/1‑285 (MQ=255)`  
                                       `gCCAAGGTCAAGCCTATTGGACACCATTGGTCGGTGCGCTAAGGCTGGTGCCGATATGTATACCGCT‑AGGAACAACGAGCACGAGACCTAGCTGATGAACGCTCTAACGAGATTATCCGTAAGCTGACCCCTGAGCAACGTCGAGAAGCTCTCAACAACGGGACCCTTCTGTATCAGGATGACCCATACGCTATGGAAGCACGCCGAGTCAAGACTGGTCGTATCGCTGCGTATCTTGTGGACGATGACGTTATGCAGCAGATAAAAGAGGGTGTCTTCCGTACTCGCGAAGAGATGGAag                                                                                                                                                                                                                                                                    <  1:669328/301‑1 (MQ=255)`  
                                                            `cacCATTGGTCGGTTCGCTAAGGCTGGTGCCGATATGTATACCGCTAAGGAACAACGAGCACGAGACCTAGCTGATGAACGCTCTAACGAGATTATCCGTAAGCTGACCCCTGAGCAACGTCGAGAAGCTCTCAACAACGGGACCCTTCTGTATCAGGATGACCCATACGCTATGGAAACACTCCGAGTCAAGACTGGTCGTAACGCTGCGTATCTTGTGGACGATGACGTTATGCAGCAGATAAAAGAGGGTGTCTTCCGTACTCGCGAAGAGATGGAAGAGTATCGCCAtctgtctctt                                                                                                                                                                                                                                                >  1:175682/1‑292 (MQ=255)`  
                                                              `ccATTGGTCGGTTCGCTAAGGCTGGTGCCGATATGTATACCGCTAAGGAACAACGAGCACGAGACCTAGCTGATGAACGCTCTAACGAGATTATCCGTAAGCTGACCCCTGAGCAACGTCGAGAAGCTCTCAACAACGGGACCCTTCTGTATCAGGATGACCCATACGCTATGGAAGCACTCCGAGTCAAGACTGGTCGTAACGCTGCGTATCTTGTGGACGATGACGTTATGCAGCAGATAAAAGAGGGTGTCTTCCGTACTCGCGAAGAGATGGAagctgtctcttatacacatctccg                                                                                                                                                                                                                                              >  1:583192/1‑279 (MQ=255)`  
                                                              `ccATTGGTCGGTTCGCTAAGGCTGGTGCCGATATGTATACCGCTAAGGAACAACGAGCACGAGACCTAGCTGATGAACGCTCTAACGAGATTATCCGTAAGCTGACCCCTGAGCAACGTCGAGAAGCTCTCAACAACGGGACCCTTCTGTATCAGGATGACCCATACGCTATGGAAGCACTCCGAGTCAAGACTGGTCGTAACGCTGCGTATCTTGTGGACGATGACGTTATGCAGCAGATAAAAGAGGGTGTCTTCCGTACTCGCGAAGAGATGGAAGAGTATCGCCATAGTCGCCTTCa                                                                                                                                                                                                                                              >  1:494978/1‑301 (MQ=255)`  
                                                              `ccATTGGTCGGTTCGCTAAGGCTGGTGCCGATATGTATACCGCTAAGGAACAACGAGCACGAGACCTAGCTGATGAACGCTCTAACGAGATTATCCGTAAGCTGACCCCTGAGCAACGTCGAGAAGCTCTCAACAACGGGACCCTTCTGTATCAGGATGACCCATACGCTATGGAAGCACTCCGAGTCAAGACTGGTCGTAACGCTGCGTATCTTGTGGACGATGACGTTATGCAGCAGATAAAAGAGGGTGTCTTCCGTACTCGCGAAGAGATGGAAGAGTATCGCCATAGTCGCCTTCa                                                                                                                                                                                                                                              >  1:449416/1‑301 (MQ=255)`  
                                                              `ccATTGGTCGGTTCGCTAAGGCTGGTGCCGATATGTATACCGCTAAGGAACAACGAGCACGAGACCTAGCTGATGAACGCTCTAACGAGATTATCCGTAAGCTGACCCCTGAGCAACGTCGAGAAGCTCTCAACAACGGGACCCTTCTGTATCAGGATGACCCATACGCTATGGAAGCACTCCGAGTCAAGACTGGTCGTAACGCTGCGTATCTTGTGGACGATGACGTTATGCAGCAGATAAAAGAGGGTGTCTTCCGTACTCGCGAAGAGATGGAAGAGTATCGCCATAGTCGCCTTCa                                                                                                                                                                                                                                              >  1:62880/1‑301 (MQ=255)`  
                                                                `aTTGGTCGGTGCGCTAAGGCTGGTGCCGATATGTATACCGCTAAGGAACAACGAGCACGAGAACTAGCGGATGAACGCTCTAACGAGATTATCCGTAAGCTGACCCCTGAACAACGTCGAGAAGCTCTCAACAACGGGACCCTTCTGTATCAGGATGACCCATACGCTATGGAAGCACTCCGAGTCAAGACTGGTCGTAACGCTGCGTATCTTGTGGACGATGACGTTATGCAGCAGATAAAAGAGGGTGTCTTCCGTACTCGCGAAGAGATGGAAGAGTATCGCCATAGTCGCCTTCAag                                                                                                                                                                                                                                            <  1:181338/301‑1 (MQ=255)`  
                                                                `aTTGGTCGATTGGCAAAGGCTGGGGCCGATGTGTATACCGCTAAGGAAAAACGGGCAAGAGACCTAGCTGATGAACGCTCTAACGAGATTATCCGTAAGCTGACCCCTGAGCAACGTCGAGAAGCTCTCAACAACGGGACCCTTCTGTAGCAGGATGACACATACGCTATGGAAGCACTCCGAGTCAAGACTGGTCGTAACGCTGCGTATCTTGTGGACGATGACGTTATGCAGCAGATAAAAGAGGGTGTCTTCCGTACTCGCGAAGAGATGGAAGAGTATCGCCATAGTCGCCTTCAag                                                                                                                                                                                                                                            <  1:359746/301‑1 (MQ=255)`  
                                                                        `gTTCGCTAAGGCTGGTGCCGATATGTATACCGCTAAGGAACAACGAGCACGAGACCTAGCTGATGAACGCTCTAACGAGATTATCCGTAAGCTGACCCCTGAGCAACGTCGAGAAGCTCTCAACAACGGGACCCTTCTGTATCAGGATTACCCATACGCTATGGAAGCACTCCGAGTCAAGACTGGTCGTAACGCTGCGTATCTTGTGGACGATGACGTTATGCAGCAGATAAAAGAGGGTGTCTTCCGTACTCGCGAAGAGATGGAAGAGTATCGCCATAGTCGCCTTCAAGAGGgcgca                                                                                                                                                                                                                                    >  1:604581/1‑300 (MQ=255)`  
                                                                           `cGCTAAGGCTGGTGCCGATATGTATACCGCTAAGGAACAACGAGCACGAGACCTAGCTGATGAACGCTCTAACGAGATTATCCGTAAGCTGACCCCTTAGCAACGTCGAGAAGCTCTCAACAACGGGACCCTTCTGTATCAGAATGACCCATACGCTATGGAAGCACTCCGAGTCAAGACTGGTCGTAACGCTGCGTATCTTGTGGACGATGACGTTATGCAGCAGATAAAAGAGGGTGTCTTCCGTACTCGCGAAGAGATGGAAGAGTATCGCCATAGTCGCCTTCAAGAGGGCGCTAAg                                                                                                                                                                                                                                 >  1:488757/1‑301 (MQ=255)`  
                                                                            `gCTAAGGCTGGTGCCGATATGTATACCGCTAAGGAACAACGAGCACGAGACCTAGCTGATGAACGCTCTAACGAGATTATCCGTAAGCTGACCCCTGAGCAACGTCGAGAAGCTCTCAACAACGGGACCCTTCTGTATCAGGATGACCCATACGCTATGGAAGCACTCCGAGTCAAGACTGGTCGTAACGCTGCGTATCTTGTGGACGATGACGTTATGCAGCAGATAAAAGAGGGTGTCTTCCGTACTCGCGAAGAGATGGAAGAGTATCGCCATAgctgtctcttatacacatctccga                                                                                                                                                                                                                                >  1:591025/1‑278 (MQ=255)`  
                                                                                 `ggCTGGTGCCGATATGTATACCGCTAAGGAACAACGAGCACGAGACCTAGCTGATGAACGCTCTAACGAGATTATCCGTAAGCTGACCCCTGAGCAACGTCGAGAAGCTCTCAACAACGGGACCCTTCTGTATCAGGATGACCCATACGCTATGGAAGCACTCCGAGTCAAGACTGGTCGTAACGCTGCGTATCTTGTGGACGATGACGTTATGCAGCAGATCAAAGAGGGTGTCTTCCGTCCTCGCGAAGAGATGGAAGAGTATCGCCCTAGTCGCATTCAAGAGGTCGCTCAGGtataa                                                                                                                                                                                                                           >  1:10616/1‑300 (MQ=255)`  
                                                                                 `ggCTGGTGCCGATATGTATACCGCTAAGGAACAACGAGCACGAGACCTAGCTGATGAACGCTCTAACGAGATTATCCGTAAGCTGACCCCTGAGCAACGTCGAGAAGCTCTCAACAACGGGACCCTTCTGTATCAGGATGACCCATACGCTATGGAAGCACTCCGAGTCAAGACTGGTCGTAACGCTGCGTATCTTGTGGACGATGACGTTATGCAGAAGATAAAAGAGGGTGTCTTCCGTACTCGCGAAGAGATGGAAGAGTATCGCCATAGTCGCCTTCAAGAGGGCGATAAGGTATAc                                                                                                                                                                                                                           >  1:117660/1‑301 (MQ=255)`  
                                                                                 `ggCTGGTGCCGATATGTATACCGCTAAGGAACAACGAGCACGAGACCTAGCTGATGAACGCTCTAACGAGATTATCCGTAAGCTGACCCCTGAGCAACGTCGAGAAACTCTCAACAACGGGACCCTTCTGTATCAGGATGACCCATACGCTATGGAAGCACTCCGAGTCAAAACTGGTCGTAACGCTGCGTATCTTGTGGACGATGACGTTATGCAGCAGATAAAAGAGGGTGTCTTCCGTACTCTCGAAGCGATGGAATATTATCGCCATAGTCGCCTTCAAGAGGGCGCTCAGGTATAc                                                                                                                                                                                                                           >  1:324371/1‑301 (MQ=255)`  
                                                                                 `gagacaggCCGATATGTATACCGCTAAGGAACAACGAGCACGAGACCTAGCTGATGAACGCTCTAACGAGATTATCCGTAAGCTGACCCCTGAGCAACGTCGAGAAGCTCTCAACAACGGGACCCTTCTGTATCAGGATGACCCATACGCTATGGAAGCACTCCGAGTCAAGACTGGTCGTAACGCTGCGTATCTTGTGGACGATGACGTTATGCAGCAGATAAAAGAGGGTGTCTTCCGTACTCGCGAAGAGATGGAAGAGTATCGCCATAGTCGCCTTCAAGAGGGCGCTAAGGTATAc                                                                                                                                                                                                                           <  1:621916/294‑1 (MQ=255)`  
                                                                                      `gTGCCGATATGTATACCGCTAAGGAACAACGAGCACGAGACCTAGCTGATGAACGCTCTAACGAGATTATCCGTAAGATGACCCCTAAGCAACGTCGAGAAGCTCTCAACAACGGGACCCTTCTGTATCAGGATGACCCATACGCTATGGAAGCACTCCGAGTCAAGACTGGTCGTAACGCTGCGTATCTTGTGGACGATGACGTTATGCAGCAGATAAAAGAGGGTGTCTTCCGTACTCGCGAAGAGATGGAAGAGTATCGCCATAGTCGCCTTCAAGAGGGCGCTAAGGTATACGCTGa                                                                                                                                                                                                                      >  1:526320/1‑301 (MQ=255)`  
                                                                                        `aacagtatGTATACCGCTAAGGAAAAACGAGCACGAGACATAGCTGAGGAAGGATCTAACGAGACTATCCGTAAGCGGCCCCCGGAGCAACGTCGAGAAGCGCTCAACAACGGGACCCGTTTGTAGCAGGATCACCCATACGGTATGGAAGCACTCCGAGTCAAGACTGGTCGTAACGTTGCTTACCTTTTGGACGATGACGTTATGCAGCTGATAAAAGAGGTCGTCTTCCGTACTCTCGAAGAGATGGAAGAGTATCGCCATAGTCGCCTTCAAGAGGGCGCTAAGGTATACGCTGAGc                                                                                                                                                                                                                    <  1:318852/296‑1 (MQ=255)`  
                                                                                           `gATATGTATACCGCTAAGGAACAACGAGCACGAGACCTAGCTGATGAACGCTCTAACGAGATTATCCGTAAGCTGACCCCTGAGCAACGTCGAGAAGCTCTCAACAACGGGACCCTTCTGTATCAGGATGACCCATACGCTATGGAAGCACTCCGAGTCAAGACTGGTCGTAACGCTGCGTATCTTGTGGACGATGACGTTATGCAGCAGATAAAAGAGGGTGTCTTCCGTACTCGCGACGAGATGGAAGAGTATCGCCATAGTCGCCTTTAAGAGGGCGCTCAGTGATACGACGAGCAgc                                                                                                                                                                                                                 >  1:380053/1‑300 (MQ=255)`  
                                                                                           `gATATGTATACCGCTAAGGAACAACGAGCACGAGACCTAGCTGATGAACGCTCTAACGAGATTATCCGTAAGCTGACCCCTGAGCAACGTCGAGAAGCTCTCAACAACGGGACCCTTCTGTATCAGGATGACCCATACGCTATGGAAGCACTCCGAGTCAAGACTGGTCGTAACGCTGCGTATCTTGTGGACGATGACGTTATGCAGCAGATAAAAGAGGGTGTCTTCCGTACTCGCGAAGAGATGGAAGAGTATCGCCATAGTCGCCTTCAAGAGGGCGCTAAGGTATACGcctgtctct                                                                                                                                                                                                                 >  1:334940/1‑293 (MQ=255)`  
                                                                                             `tatGTATACCGCTAAGGAACAACGAGCACGAGACCTAGCTGATGAACGCTCTAACGAGATTATCCGTAAGCTGACCACTGAGCAACGTCGAGAAGCTCTCAACAACGGGACCCTTCTGTATCAGGATGACCCATACGCTATGGAAGCACTCCGAGTCAAGACTGGTCGTAACGCTGCGTATCTTGTGGACGATGACGTTATGCAGCAGATAAACGAGGATGTCTTCCGTACTCGCGAAGAGATGAAAGAGTATGGCCATAGTAGCCTTACAGAGGGCGCTAGGGTATACGCTGAGCAGTTc                                                                                                                                                                                                               >  1:483712/1‑301 (MQ=255)`  
                                                                                             `tatGTAGACCGCTACTGCACCATGAGCACGGGCCCTCGCTAATGAACGCTCGAACGCCATTATCCGTAGTCTGACACCTGAGCAATGTCGAGAAGTTCTCAACAACTGGACCCTCCTGTATCAGGATGACCTATCCGCTATGGAATCACTCCGAGTCAAGCCTGGTCGTACTGCTGCGTATATTGTGGACGATTACGTTATTCAGCAGATAAAAGAGGGTGTCGTCCGTACTCGCGAAGAGATGGAAGAGTATCGCCATAGTCGCCTTCAAGAGTGCGCTAAGGTATACGCTGAGCATTTc                                                                                                                                                                                                               <  1:255961/301‑1 (MQ=255)`  
                                                                                                `gTATACCGCTAAGGAACAACGAGCACGAGACCTAGCTGATGAACGCTCTAACGAGATTATCCGTAAGCTGACCCCTGAGCAACGTCGAGAAGCTCTCAACAACGGGACCCTTCTGTATCAGGATGACCCATACGCTATGGAAGCACTCCGAGTCAAGACTGGTCGTAACGCTGCGTATCTTGTGGACGATGACGTTATGCAGCAGATAAAAGAGGGTGTCTTCCGTACTCGCGAAGCGATGGAAGAGTATCGCCATAGTCGCCTTCAAGAGGGCGCTAAGGTATACGCTGAGCAGTTCGGc                                                                                                                                                                                                            >  1:266722/1‑301 (MQ=255)`  
                                                                                                  `ataCCGCTAAGGAACAACGAGCACGAGACCTAGCTGATGAACGCTCTAACGAGATTATCCGTAAGCTGACCCCTGAGCAACGTCGAGAAGCTCTCAACAACGGGACCCTTCTGTATCAGGATGACCCATACGCTATGGAAGCACTCCGAGTCAAGACTGGTCGTAACGCTGCGTATCTTGTGGACGATGACGTTATGCAGCAGATAAAAGAGGGTGTCTTCCGTACTCGCGAAGAGATGGAAGAGTATCGCCATAGTCGCCTTCAAGAGGGCGCTAAGGTACACGCTGAGAAGTTCGGCAt                                                                                                                                                                                                          <  1:228511/301‑1 (MQ=255)`  
                                                                                                        `ttgtatcagagacggcccgcgaCCTAGCTGATTAACGCTCTAACGAGAGTATCCGGAAGCTGACCCCTGAGCAACGTCGAGCAGCTCTCAACAACGGGACCCTTCTGTATCAGGATGACCCATACGCTATGGAAGCACTCCGAGTCAAGACTGGTCGTAACGCTGCGTATCTTGTGGACGAAGACGTTATGCAGCAGATAAAAGAGGGTGTCTTCCGTACTCGCGAAGAGATGGAAGAGTATCGCCATAGTCGCCTTCAAGAGGGCGCTAAGGTATACGCTGAGCAGTTCGGCATCGAccc                                                                                                                                                                                                    <  1:410225/281‑1 (MQ=255)`  
                                                                                                                      `gCACGAGACCTAGCTGATGAACGCTCTAACGAGATTATCCGTAAGCTGACCCCTGAGCAACGTCGAGAAGCTCTCAACAACGGGACCCTTCTGTATCAGGATGACCCATACGCTATGGAAGCACTCCGAGTCAAGACTGGTCGTAACGCTGCGTATCTTGTGGACGATGACGTTATGCAGCAGATAAAAGAGGGTGTCTTCCGTACTCGCGAAGAGATGGAAGAGTATCGCCATAGTCGCCTTCAAGAGGGCGCTAAGGTATACGCTGAGCAGTTCGGCATCGACCCTGAGGACGTTGAtt                                                                                                                                                                                      >  1:639951/1‑301 (MQ=255)`  
                                                                                                                           `agaCCTAGCTGATGAACGCTCTAACGAGATTATCCGTAAGCTGACCCCTGAGCAACGTCGAGAAGCTCTCAACAACGGGACCCTTCTGTATCAGGATGACCCATACGCTATGGAAGCACTCCGAGTCAAGACTGGTCGTAACGCTGCGTATCTTGTGGACGATGACGTTATGCAGCAGATAAAAGAGGGTGTCTTCCGTACTCGCGAAGAGATGGAAGAGTATCGCCATAGTCGCCTTCAAGAGGGCGCTAAGGTATACGCTGAGCAGTTCGGCATCGACCCTGAGGACGTTGATTATCAg                                                                                                                                                                                 >  1:211178/1‑301 (MQ=255)`  
                                                                                                                                  `gCTGATGAACGCTCTAACGAGATTATCCGTAAGCTGACCCCTGAGCAACGTCGAGAAGCTCTCAACAACGGGACCCTTCTGTATCAGGATGACCCATACGCTATGGAAGCACTCCGAGTCAAGACTGGTCGTAACGCTGCGTATCTTGTGGACGATGACGTTATGCAGCAGATAAAAGAGGGTGTCTTCCGTACTCGCGAAGAGATGGAAGAGTATCGCCATAGTCGCCGTCAAGAGGGTGCGAAGGTATACGCTGAGCAGTTCGGCATCGACCCTGAGGACGTTGATGATCAGCGTGGtg                                                                                                                                                                          >  1:500525/1‑300 (MQ=255)`  
                                                                                                                                     `gatgaACGCTCTAACGAGATTATCCGTAAGCTGACCCCTGAGCAACGTCGAGAAGCTCTCAACAACGGGACCCTTCTGTATCAGGATGACCCATACGCTATGGAAGCACTCCGAGTCAAGACTGGTCGTAACGCTGCGTATCTTGTGGACGATGACGTTATGCAGCAGATAAAAGAGGGTGTCTTCCGTACTCGCGAAGAGATGGAAGAGTATCGCCATAGTCGCCTTCAAGAGGGCGCTAAGGTATACGCTGAGCAGTTCGGCATCGACCCTGAGGACGTTGATTATCAGctgtctcttt                                                                                                                                                                       >  1:217657/1‑292 (MQ=255)`  
                                                                                                                                        `gaACGCTCTAACGAGATTATCCGTAAGCTGACCCCTGAGCAACGTCGAGAAGCTCTCAACAACGGGACCCTTCTGTATCAGGATGACCCATACGCTATGGAAGCACTCCGAGTCAAGACTGGTCGTAACGCTGCGTATCTTGTGGACGATGACGTTATGCAGCAGATAAAAGAGGGTGTCTTCCGTACTCGCGAAGAGATGGAAGAGTATCGCCATAGTCGCCTTCAAGAGGGCGCTAAGGTATACGCTGAGCAGTTCGGAATCGACCCTGAGGACGTTGATTATCAGCGTGGTTTCAACg                                                                                                                                                                    <  1:274039/301‑1 (MQ=255)`  
                                                                                                                                            `gagatgtgtataagagacagaaGCTGACCCCTGAGCAACGTCGAGAAGCTCTCAACAACGGGACCCTTCTGGATCAGGATGACCCATACGCTATGGAAGCACTCCGAGTCAAGACTGGTCGTAACGCTGCGTATCTTGTGGACGATGACGTTATGCAGCAGATAAAAGAGGGTGTCTTCCGTACTCGCGAAGAGATGGAAGAGTATCGCCATAGTCGCCTTCAAGAGGGCGCTAAGGTATACGCTGAGCAGTTCGGCATCGACCCTGAGGACGTTGATTATCAGCGTGGTTTCAACGGGGa                                                                                                                                                                <  1:92865/281‑1 (MQ=255)`  
                                                                                                                                            `gCTCTAACGAGATTATCCGTAAGCTGACCCCTGAGCAACGTCGAGAAGCTCTCAACAACGGGACCCTTCTGTATCAGGATGACCCATACGCTATGGAAGCACTCCGAGTCAAGACTGGTCGTAACGCTGCGTATCTTGTGGACGATGACGTTATGCAGCAGATAAAAGAGGGTGTCTTCCGTACTCGCGAAGAGATGGAAGAGTATCGCCATAGTCGCCTTCAAGAGGGCGCTAAGGTATACGCTGAGCAGTTCGGCATCGACCCTGAGGACGTTGATTATCAGCGTGGTTTCAACGGGGa                                                                                                                                                                <  1:13663/301‑1 (MQ=255)`  
                                                                                                                                               `atAACGAGGTTATCCGTAAGCTGACCCCTGGGCAACGTCGATAAGACCTAAACAACGGGACCCTTCTTTATCCGGATAACCCATACGCTATGGAAGAACTCCGGTTCAAGACTGGTCGTAACCCTGCGTATCTTGGGGACGATGACGTTATGCAGCAGATAAAAGAGGGTGTCTTCCGTACTCGCGAAGAGATGGAAGAGTATCGCCATAGTCGCCTTCAAGAGGGCGCTAAGGTATACGCTGAGCAGTTCGGCATCGACCCTGAGGACGTTGATTATCAGCGTGGTTTCAACGGGGACAt                                                                                                                                                             <  1:610480/300‑1 (MQ=255)`  
                                                                                                                                                  `ggagaagtgtataagagacagccccTGAGCAACGTCGAGAAGCTCTCAACAACGGTACCCTTCTGTATCATGATGACCCATACGCTATGGAAGCACTCCGAGTCAAGACTGGTCGTAACGCTGCGTATCTTGTGGACGATGACGTTATGCAGCAGATAAAAGAGGGTGTCTTCCGTACTCGCGAAGAGATGGAAGAGTATCGCCATAGTCGCCTTCAAGAGGGCGCTAAGGTATATGCTGAGCAGTTCGGCATCGACCCTGAGGACGTTGATTATCAGCGTGGTTTCAACGGGGACATTAc                                                                                                                                                          <  1:557371/280‑1 (MQ=255)`  
                                                                                                                                                     `agaTTATCCGTAAGCTGACCCCTGAGCAACGTCGAGAAGCTCTCAACAACGGGACCCTTCTGTATCAGGATGACCCATACGCTATGGAAGCACTCCGAGTCAAGACTGGTCGTAACGCTGCGTATCTTGTGGACGATGACGTTATGCAGCAGATAAAAGAGGGTGTCTTCCGTACTCGCGAAGAGATGGAAGAGTATCGCCATAGTCGCCTTCAAGAGGGCGCTAAGGTATACGCTGAGCAGTTCGGCATCGACCCTGAGGACGTTGATTATCAGCGTGGTTTCAACGGGGACATTACCGa                                                                                                                                                       >  1:455973/1‑301 (MQ=255)`  
                                                                                                                                                       `aTTATCCGTAAGCTGACCCCTGAGCAACGTCGAGAAGCTCTCAACAACGGGACCCTTCTGTATCAGGATGACCCATACGCTATGGAAGCACTCCGAGTCAAGACTGGTCGTAACGCTGCGTATCTTGTGGACGATGACGTTATGCAGCAGATAAAAGAGGGTGTCTTCCGTACTCGCGAAGAGATGGAAGAGTATCGCCATAGTCGCCTTCAAGAGGGCGCTAAGGTATACGCTGAGCAGTTCGGCATCGACCCTGAGGACGTTGATTATCAGCGTGGTTTCAACGGGGACATTAcctgtc                                                                                                                                                     >  1:597260/1‑297 (MQ=255)`  
                                                                                                                                                         `tATCCGTAAGCTGACCCCTGAGCAACGTTGAGAAGCGCTCAAAAACGTGTCCATTCTGTATCAGGATGACCCATACGCTATGGAAGCACTACGAGTCAAGACTGGTCGTAACGCTGCGTATCTTGTGGACGATGACGTTATGCAGCAGATAAAAGAGGGTGTCTTCCGTACTCGCGAAGAGATGGAAGAGTATCGCCATAGTCGCCTTCAAGAGGGCGCTAAGGTATACGCTGAGCAGTTCGGCATCGACCCTGAGGACGTTGATTATCAGCGTGGTTTCAACGGGGACATTACCGAGCGt                                                                                                                                                   <  1:260118/301‑1 (MQ=255)`  
                                                                                                                                                           `tCCGTAAGCTGACCCCTGAGCAACGTCGAGAAGCTCTCAACAACGGGACCCTTCTGTATCAGGATGACCCATACGCTATGGAAGCACTCCGAGTCAAGACTGGTCGTAACGCTGCGTATCTTGTGGACGATGACGTTATGCAGCAGATAAAAGAGGGTGTCTTCCGTACTCGCGAAGAGATGGAAGAGTATCGCCATAGTCGCCTTCAAGAGGGCGCTAAGGTATACGCTGAGCAGTTCGGCATCGACCCTGAGGACGTTGATTATCAGCGTGGTTTCAACGGGGACATTACCGAGCGtct                                                                                                                                                 >  1:564841/1‑299 (MQ=255)`  
                                                                                                                                                           `tCCGTAAGCTGACCCCTGAGCAACGTCGAGAAGCTCTCAACAACGGGACCCTTCTGTATCAGGATGACCCATACGATATGGAAGCACTCCGAGTCAAGACTGGTCGTAACGCTGCGTATCTTGTGGACGATGACGTTATGCAGCAGATAAAAGAGGGTGTCTTCCGTACTCGCGAAGAGATGGAAGAGTATCGCCATAGTCGCCTTCAAGAGGGCGCTAAGGTATACGCCGAGCAGTTCGGCACCGACCCTGAGGACGTGGATTATCAGCGTGGTTTCAAAggccgtctcttattcacatt                                                                                                                                                 >  1:245196/1‑283 (MQ=255)`  
                                                                                                                                                            `ccGTAAGCTGACCCCTGAGCAACGTCGAGAAGCTCTCAACAACGGGACCCTTCTGTATCAGGATGACCCATACGCTATGGAAGCACTCCGAGTCAAGACTTGTCGTAACGCTGCGTATCTTGTGGACGATGACGTTATGCAGCAGATAAAAGAGGGTGTCTTCCGTACTCGCGAAGAGATGGAAGAGTATCGCCATAGTCGCCTTCAAGAGGGCGCTAAGGTATACGCTGAGCAGTTCGGCATCGACCCTGAGGACGTTGATTATCAGCGTGGTTTCAACGGGGACATTACCGAGCGTAAc                                                                                                                                                <  1:123694/301‑1 (MQ=255)`  
                                                                                                                                                              `ggagatgggtataagagacaggTCGAGAAGCTCTCAACAACGGGACCCTTCTGTATCAGGATGACCCATACGCTATGGAAGCACTCCGAGTCAAGACTGGTCGTAACGCTGCGTATCTTGTGGACGATGACGTTATGCAGCAGATAAAAGAGGGTGTCTTCCGTACTCGCGAAGAGATGGAAGAGTATCGCCATAGTCGCCTTCAAGAGGGCGCTAAGGTATACGCTGAGCAGTTCGGCATCGACCCTGAGGACGTTGATTATCAGCGTGGTTTCAACGGGGACATTACCGAGCGTAACAt                                                                                                                                              <  1:28875/280‑1 (MQ=255)`  
                                                                                                                                                              `gTAAGCTGACCCCTGAGCAACGTCGAGAAGCTCTCAACAACGGGACCCTTCTGTATCAGGATGACCCATACGCTATGGAAGCACTCCGAGTCAAGACTGGTCGTAACGCTGCGTATCTTGTGGACGATGACGTTATGCAGCAGATAAAAGAGGGTGTCTTCCGTACTCGCGAAGAGATGGAAGAGTATCGCCATAGTCGCCTTCAAGAGGGCGCTAAGGTATACGCTGAGCAGTTCGGCATCGACCCTGAGGACGTTGATTATCAGCGTGGTTTCAACGGGGACATTACCGAGCGTAACAt                                                                                                                                              <  1:422467/301‑1 (MQ=255)`  
                                                                                                                                                              `atAAGCTGACCCCTGAGCAACTGCGAGAAGCTCTCAACAACGGGACCCTTCTGTATCAGGATGACCCATACGCTATGGAAGCACTCCGAGTCAAGACTGGTCGTAACGCTGCGTATCTTGTGGACGATGACGTTATGCAGCAGATAAAAGAGGGTGTCTTCCGTACTCGCGAAGAGATGGAAGAGTATCGCCATAGTCGCCTTCAAGAGGGCGCTAAGGTATACGCTGAGCAGTTCGGCATCGACCCTGAGGACGTTGATTATCAGCGTGGTTTCAACGGGGACATTACCGAGCGTAACAt                                                                                                                                              <  1:658149/300‑1 (MQ=255)`  
                                                                                                                                                                     `gACCCCTGAGCAACGTCGAGAAGCTCTCAACAACGGGACCCTTCTGTATCAGGATGACCCATACGCTATGGAAGCACTCCGAGTCAAGACTGGTCGTAACGCTGCGTATCTTGTGGACGATGACGTTATGCAGCAGATAAAAGAGGGTGTCTTCCGTACTCGCGAAGAGATGGAAGAGTATCGCCATAGTCGCCTTCAAGAGGGCGCTAAGGTATACGCTGAGCAGTTCGGCATCGACCCTGAGGACGTTGATTATCAGCGTGGTTTCAACGGGGACATTACCGAGCGTAACATCTCGCTg                                                                                                                                       <  1:294474/301‑1 (MQ=255)`  
                                                                                                                                                                          `cggagatgtgtataagagacagcaccaacGGGACCCTTCTGGATCAGGATGACCCATACGCTATGGAAGCACTCCGAGTCAAGACTGGTCGTAACGCTGCGTATCTTGTGGACGATGACGTTATGCAGCAGATAAAAGAGGGTGTCTTCCGTACTCGCGAAGAGATGGAAGAGTATCGCCATAGTCGCCTTCAAGAGGGCGCTAAGGTATACGCTGAGCAGTTCGGCATCGACCCTGAGGACGTTGATTATCAGCGTAGTTTCAACGGGGACATTACCGAGCGTAACATCTCGCTGTATgg                                                                                                                                  <  1:443493/276‑1 (MQ=255)`  
                                                                                                                                                                          `cTGAGCAACGTCGAGAAGCTCTCAACAACGGGACCCTTCTGTATCAGGATGACCCATACGCTATGGAAGCACTCCGAGTCAAGACTGGTCGTAACGCTGCGTATCTTGTGGACGATGACGTTATGCAGCAGATAAAAGAGGGTGTCTTCCGTACTCGCGAAGAGATGGAAGAGTATCGCCATAGTCGCCTTCAAGAGGGCGCTAAGGTATACGCTGAGCAGTTCGGCATCGACCCTGAGGACGTTGATTATCAGCGTGGTTTCAACGGGGACATTACCGAGCGTAACATCTCGCTGTATgg                                                                                                                                  <  1:149413/301‑1 (MQ=255)`  
                                                                                                                                                                                   `gTCGAGAAGCTCTCAACAACGGGACCCTTCTGTATCAGGATGACCCATACGCTATGGAAGCACTCCGAGTCAAGACTGGTCGTAACGCTGCGTATCTTGTGGACGATGACGTTATGCAGCAGATAAAAGAGGGTGTCTTCCGTACTCGCGAAGAGATGGAAGAGTATCGCCATAGTCGCCTTCAAGTGGGCGCTAAGGTATACGCTGAGCAGTTCGGCATCGACCCTGAGGACGTTGATTACCAGCGTGGTTTCAACGGGGACATTACCGAGCGTAAAATCTCGCTGTATggctgtctctt                                                                                                                         >  1:129999/1‑292 (MQ=255)`  
                                                                                                                                                                                    `cagatcaGCTCTCAACAGCGGGACCCTTCTGGATCAGGATGACCCATACGATATGGAAGAACTCCGAGTCAAGACTGGTCGTAACGCAGCGTATCTTGTGGACGATGACGTTATGCAGCAGATAAAAGAGGGTGTCTTCCTGACTCGCGAAGAGATGTAAGAGTATCGCCATAGTCGCCTTCAAGAGGGCGCTAAGGTATACGCTGAGCAGTTCGGCATCGACCCTGAGGACGTTGATTATCAGCGTGGTTTCAACGGGGACATTACCGAGCGTAACATCTCGCTGTATGGTGCGCATGAt                                                                                                                        <  1:644800/295‑1 (MQ=255)`  
                                                                                                                                                                                         `atggtcccaacaaCCGGACCCTTCCGTATCAGGCTGACCCCACCGATATAGAAGCACTCCGAGTCAAGACTGGTCGTAACGCTGCGTATCTTGTGGACGATGACGTTATGCAGCCGATAAAAGAGGGTGTCTTCCGTACTCGCGGAGCGATGGAACGTTATAGCCATAGTCGCCTTCAAGAGGGCGCTAAGGTATACGCTGAGCAGTTCGGCATCGACCCTGAGGACGTTGATTATCAGCGTGGTTTCAACGGGGACATTACCGAGCGTAACATCTCGCTGTATGGTGCGCATGATAActt                                                                                                                   <  1:506622/294‑1 (MQ=255)`  
                                                                                                                                                                                           `gCTCTCAACAACGGGACCCTTCTGTATCAGGATGACCCATACGCTATGGAAGCACTCCGAGTCAAGACTGGTCGTAACGCTGCGTATCTTGTGGACGATGACGTTATGCAGCAGATAAAAGAGGGTGTCTTCCGTACTCGCGAAGAGATGGAAGAGTATCGCCATAGTCGCCTTCAAGAGGGCGCTAAGGTATACGCTGAGCAGTTCGGCATCGACCCTGAGGACGTTGATTATCAGCGTGGTTTCAACGGGGACATTACCGAGCGTAACATCTCTCTGTATGGTGCGCATGATAActtct                                                                                                                 >  1:238720/1‑301 (MQ=255)`  
                                                                                                                                                                                           `gCTCTCAACAACGGGACCCTTCTGTATCAGGATGACCCATACGCTATGGAAGCACTCCGAGTCAAGACTGGTCGTAACGCTGCGTATCTTGTGGACGATGACGTTATGCAGCAGATAAAAGAGGGTGTCTTCCGTACTCGCGAAGAGATGGAAGAGTATCGCCATAGTCGCCTTCAAGAGGGCGCTAAGGTATACGCTGAGCAGTTCGGCATCGACCCTGAGGACGTTGATTATCAGCGTGGTTTCAACGGGGACATTACCGAGCGTAACATCTCGCTGTATGGTGCGCATGATAtcttct                                                                                                                 >  1:616267/1‑301 (MQ=255)`  
                                                                                                                                                                                                     `acGGGACCCTTCTGTATCAGGATGACCCATACGCTATGGAAGCACTCCGAGTCAAGACTGGTCGTAACGCTGCGTATCTTGTGGACGATGACGTTATGCAGCAGATAAAAGAGGGTGTCTTCCGTACTCGCGAAGAGATGGAAGAGTATCGCCATAGTCGCCTTCAAGAGGGCGCTAAGGTATACGCTGAGCAGTTCGGCATCGACCCTGAGGACGTTGATTATCAGCGTGGTTTCAACGGGGACATTACCGAGCGTAAAATCTCGCTGTATGGTGCGCATGATAACTTCTTGAGCCAGCa                                                                                                       >  1:461344/1‑301 (MQ=255)`  
                                                                                                                                                                                                           `cccTTCTGTATCAGGATGACCCATACGCTATGGAAGCACTCCGAGTCAAGACTGGTCGTAACGCTGCGTATCTTGTGGACGATGACGTTATGCAGCAGATAAAAGAGGGTGTCTTCCGTACTCGCGAAGAGATGGAAGAGTATCGCCATAGTCGCCTTCAAGAGGGCGCTAAGGTATACGCTGAGCAGTTCGGCATCGACCCTGAGGACGTTGATTATCAGCGT‑GTTTCAACGGGGACATTACCGAGCGTAACATCTCGCTGTATGGTGCGCATGATAACTTCTTGAGCCAGCAAGCTCAg                                                                                                >  1:346589/1‑301 (MQ=255)`  
                                                                                                                                                                                                            `ccTTCTGTATCAGGATGACCCATACGCTATGGAAGCACTCCGAGTCAAGACTGGTCGTAACGCTGCGTATCTTGTGGACGATGACGTTATGCAGCAGATAAAAGAGGGTGTCTTACGTACTCGCGAAGAGATGTAAGAGTATCGCCATAGTCGCCTTCAAGAGGGCGCTAAGGTATACGCTGAGCAGTTCGGCATCGACCCTGAGGACGTTGATTATCAGCGTGGTTTCAACGGGGACATTACCGAGCGTAACATCTCGCTGTATGGTGCGCATGATAACTTCTTGAGCCAGCAAGCTCAg                                                                                                >  1:161422/1‑301 (MQ=255)`  
                                                                                                                                                                                                               `tCTGTATCAGGATGACCCATACGCTATGGAAGCACTCCGAGTCAAGACTGTTCGTAACGCTGCGTATCTTGTGGACGTTGACGTTATGCAGCAGATAAAAGAGGGTGTCTTCCGTACTCGCGAATAGATGGAATAGTATCGCCATAGTCGCCTTCAAGAGGTCGCTAAGGTATACGCTGAGCAGTTCGGCATCGACCCTGAGGACGTTGATTATCAGCGTGGTTTCAACTGGGACATTACCGAGCGTAACATCTCGCTGTATGGTGCGCATTATAACTTCTTGAGCCAGCAAGCTCAGAAg                                                                                             <  1:199680/301‑1 (MQ=255)`  
                                                                                                                                                                                                               `tCTGTATCAGGATGACCCATACGCTATGGAAGCACTCCGAGTCAAGACTGGTCGTAACGCTGCGTATCTTGTGGACGATGACGTTATGCAGCAGATAAAAGAGGGTGTCTTCCGTACTCGCGAAGAGATGGAAGAGCATCGCCATAGTCGCCTTCAAGAGGGCGCTAAGGTATACGCTGAGCAGTTCGGCATCGACCCTGAGGACGTTGATTATCAGCGTGGTTTCAACGGGGACATTACCGAGCGTAACATCTCGCTGTATGGTGCGCATGATAACTTCTTGAGCCAGCAAGCTCAGAAg                                                                                             <  1:31281/301‑1 (MQ=255)`  
                                                                                                                                                                                                               `agggacagaGGATGACCCATACGCTATGGAAGCACTCCGAGTCAAGACTGGTCGTAATGCTGCGTATCTTGTGGACGATGACGTTATGCAGCAGATAAAAGAGGGTGTCTTCCGTACTCGCGAAGAGATGGAAGAGTATCGCCATAGTCGCCTTCAAGAGGGCGCTAAGGTATACGCTGAGCAGTTCGGCATCGACCCTGAGGACGTTGATTATCAGCGTGGTTTCAACGGGGACATTACCGAGCGTAACATCTCGCTGTATGGTGCGCATGATAACTTCTTGAGCCAGCAAGCTCAGAAg                                                                                             <  1:571225/293‑1 (MQ=255)`  
                                                                                                                                                                                                                  `gTATCAGGATGACCCATACGCTATGGAAGCACTCCGAGTCAAGACTGGTCGTAACGCTGCGTATCTTGTGGACGATGACGTTATGCAGCAGATAAAAGAGGGTGTCTTCCGTACTCGCGAAGAGATGGAAGAGTATCGCCATAGTCGCCTTCAAGAGGGCGCTAAGGTATACGCTGAGCAGTTTGGCATCGACCCTGAGGACGTTGATTATCAGCGTGGTTTCAACGGGGACATTCCCGAGCGTAACCTCTCGCTGTTTTGTGCGCATGTTAACTTCTTGAGCCAGCAAGCTCAGAAgggg                                                                                          >  1:250646/1‑300 (MQ=255)`  
                                                                                                                                                                                                                  `gTATCAGGATGACCCATACGCTATGGAAGCACTCCGAGTCAAGACTGGTCGTAACGCTGCGTATCTTGTGGACGATGACGTTATGCAGCAGATAAAAGAGGGTGTCTTCCGTACTCGCGAAGAGATGGAAGAGTATCGCCATAGTCGCCTTCAAGAGGGCGCTAAGGTATACGCTGAGCAGTTCGGCATCGACCCTGAGGACGTTGATTATCAGCGTGGTTTCAACGGGGACATTACCGAGCGTAACATCTCGCTGTATGGTGCGCATGATAACTTCTTGAGCCAGCAAGCTCAGAAGGgc                                                                                          >  1:186746/1‑301 (MQ=255)`  
                                                                                                                                                                                                                  `gTATCAGGATGACCCATACGCTATGGAAGCACTCCGAGTCAAGACTGGTCGTAACGCTGCGTATCTTGTGGACGATGACGTTATGCAGCAGATAAAAGAGGGTGTCTTCCGTACTCGCGAAGAGATGGAAGAGTATAGCCATAGTCGCCTTCAAGAGGGCGCTAAGGTATACGCTGAGCAGTTCGGCATCGACCCTGAGGACGTTGATTATCAGCGTGGTTTCAACGGGGACATTACCGAGCGTAACATCTCGCTGTATGGTGCGCATGATAACTTCTTGAGCCAGCAAGCTCAGAAgctg                                                                                          >  1:111854/1‑298 (MQ=255)`  
                                                                                                                                                                                                                   `tATCAGGATGACCCATACGCTATGGAAGCACTCCGAGTCAAGACTGGTCGTAACGCTGCGTATCTTGTGGACGATGACGTTATGCAGCAGATAAAAGAGGGTGTCTTCCGTACTCGCGAAGAGATGGAAGAGTATCGCCATAGTCGCCTTCAAGAGGGCGCTAAGGTATACGCTGAGCAGTTCGGCATCGACCCTGAGGACGTTGATTATCAGCGTGGTTTCAACGGGGACATTACCGAGCGTAACATCTCGCTGTATGGTGCGCATGATAACTTCTTGAGCCAGCAAGCTCAGAAGGgcg                                                                                         <  1:437318/301‑1 (MQ=255)`  
                                                                                                                                                                                                                      `cAGGATGACCCATACGCTATGGAAGCACTCCGAGTCAAGACTGGTCGTAACGCTGCGTATCTTGTGGACGATGACGTTATGCAGCAGATAAAAGAGGGTGTCTTCCGTACTCGCGAAGAGATGGAAGAGTATCGCCATAGTCGCCTTCAAGAGGGCGCTAAGGTATACGCTGAGCAGTTCGGCATCGACCCTGAGGACGTTGATTATCAGCGTGGTTTCAACGGGGACATTACCGAGCGTAACATCTCGCTGTATGGTGCGCATGATACCTTCTTGAGCCAGCAAGCTCAGAAGGGCGCTa                                                                                      >  1:386575/1‑301 (MQ=255)`  
                                                                                                                                                                                                                       `aGGATGACCCATACGCTATGGAAGCACTCCGAGTCAAGACTGGTCGTAACGCTGCGTATCTTGTGGACGATGACGTTATGCAGCAGATAAAAGAGGGTGTCTTCCGTACTCGCTAAGAGATGGAAGAGTATCGCCATAGTCGCCTTCAAGAGGGCGCTAAGGTATACGCTGAGCAGTTCGGCATCGACCCTGAGGACGTTGATTATCAGCGTGGTTTCAACGGGGACATTACCGAGCGTAACATCTCGCTGTATGGTGCGCATGATAACTTCTTGAGCCAGCAAGCTCAGAAGGGCGCTAt                                                                                     <  1:167780/301‑1 (MQ=255)`  
                                                                                                                                                                                                                       `aGGATGACCCATACGCTATGGAAGCACTCCGAGTCAAGACTGGTCGTAACGCTGCGTATCTTGTGGACGATGACGTTATGCAGCAGATAAAAGAGGGTGTCTTCCGTACTCGCGAAGAGATGGAAGAGTATCGCCATAGTCGCCTTCAAGAGGGCGCTAAGGTATACGCTGAGCAGTTCGGCATCGACCCTGAGGACGTTGATTATCAGCGTGGTTTCAACGGGGACATTACCGAGCGTAACATCTCGCTGTATGGTGCGCATGATAACTTCTTGAGCCAGCAAGCTCAGAAGGGCGCTAt                                                                                     <  1:192675/301‑1 (MQ=255)`  
                                                                                                                                                                                                                        `ggATGACCCATACGCTATGGAAGCACTCCGAGTCAAGACTGGTCGTAACGCTGCGTATCTTGTGGACGATGACGTTATGCAGCAGATAAAAGAGGGTGTCTTCCGTACTCGCGAAGAGATGGAAGAGTATCGCCATAGTCGCCTTCAAGAGGGCGCTAAGGTATACGCTGAGCAGTTCGGCATCGACCCTGAGGACGTTGATTATCAGCGTGGTTTCAACGGGGACATTACCGAGCGTAACATCTCGCTGTATGGTGCGCATGATAACTTATTGAGCCAGCAAGCTCAGAAGGGCGCTATc                                                                                    >  1:35263/1‑301 (MQ=255)`  
                                                                                                                                                                                                                              `cccgaaagcgaTGGAAGCACTCGGATTCAAGACTGGTCGTAACGCTGCGTATCTTTTGGACGATTACGTTAGGCAGCAGATAAAAGAGGGTGTCTTCCGTACTCGCGAAGAGATGGAAGAGTATCGAAATAGTCTCCTTCAAGAGGGCGCTAAGGTATACGCTGAGCAGTTCGGCATCGACCCTGAGGACGTTGATTATCAGCGTGGTTTCAACGGGGACATTACCGAGCGTAACATCTCGCTGTATGGTGCGCATGATAACTTCTTGAGCCAGCAAGCTCAGAAGGGCGCTATCATGAAc                                                                              <  1:299907/291‑1 (MQ=255)`  
                                                                                                                                                                                                                              `cccATACGCTATGGAAGCACTCCGAGTCAAGACTGGTCGTAACGCTGCGTATCTTGTGGACGATGACGTTATGCAGCAGATAAAAGAGGGTGTCTTCCGTACTCGCGAAGAGATGGAAGAGTATCGCCATAGTCGCCTTCAAGAGGGCGCTAAGGTATACGCTGAGCAGTTCGGCATCGACCCTGAGGACGTTGATTATCAGCGTGGTTTCAACGGGGACATTACCGAGCGTAACATCTCGCTGTATGGTGCGCATGATAACTTCTTGAGCCAGCAAGCTCAGAAGGGCGCTATCATGaaa                                                                              >  1:36986/1‑300 (MQ=255)`  
                                                                                                                                                                                                                              `cccATACGCTATGGAAGCACTCCGAGTCAAGACTGGTCGTAACGCTGCGTATCTTGTGGACGATGACGTTATGCAGCAGATAAAAGAGGGTGTCTTCCGTACTCGCGAAGAGATGGAAGAGTATCGCCATAGTCGCCTTCAAGAGGGCGCTAAGGTATACGCTGAGCAGTTCGGCATCGACCCTGAGGACGTTGATTATCAGCGTGGTTTCAACGGGGACATTACCGAGCGTAACATCTCGCTGTATGGTGCGCATGATAACTTCGTGAGCCAGCAAGCTCAGAAGGGCGCTAACATGAAc                                                                              >  1:2047/1‑301 (MQ=255)`  
                                                                                                                                                                                                                              `cccATACGCTATGGAAGCACTCCGAGTCAAGACTGGTCGTAACGCTGCGTATCGTGTGGACGATGACGTTATGCAGCAGATAAAAGAGGGTGTCTTCCGTACTCGCGAAGAGATGGAAGAGTATCGCCATAGTCGCCTTCAAGAGGGCGCTAAGGTATACGCTGAGCAGTTCGGCATCGACCCTGAGGACGTTGATTATCAGCGTGGTTTCAACGGGGACATTACCGAGCGTAACATCTCGCTGTATGGTGCGCATGATAACTTCTTGAGCCAGCAAGCTCAGAAGGGCGCTATCATGAAc                                                                              <  1:478487/301‑1 (MQ=255)`  
                                                                                                                                                                                                                                      `cTATGGAAGCACTCCGAGTCAAGACTGGTCGTAACGCTGCGTATCTTGTGGACGATGACGTTATGCAGCAGATAAAAGAGGGTGTCTTCCGTACTCGCGAAGAGATGGAAGAGTATCGCCATAGTCGCCTTCAAGAGGGCGCTAAGGTATACGCTGAGCAGTTCGGCATCGACCCTGAGGACGTTGATTATCAGCGTGGTTTCAACGGGGACATTACCGAGCGTAACATCTCGCTGTATGGTGCGCATGATAACTTCTTGAGCCAGCAAGCTCCGAAGGGCGCTATCATGAACAGCCGAGt                                                                      >  1:404183/1‑301 (MQ=255)`  
                                                                                                                                                                                                                                      `cTATGGAAGCACTCCGAGTCAAGACTGGTCGTAACGCTGCGTATCTAGTGGACGATGACGTTATGCAGCAGATAAAAGAGGGTGTCTTCCGTACTCGCGAAGAGATGGAAGAGTATCGCCATAGTCGCCTTCAAGAGGGCGCTAAGGTATACGCTGAGCAGTTCGGCATCGACCCTGAGGACGTTGATTATCAGCGTGGTTTCAACGGGGACATTACCGAGCGTAACATCTCGCTGTATGGTGCGCATGATAACTTCTTGAGCCAGCAAGCTCAGAAGGGCGCTATCATGAACAGCCGAGt                                                                      >  1:550341/1‑301 (MQ=255)`  
                                                                                                                                                                                                                                           `gAAGCACTCCGAGTCAAGACTGGTCGTAACGCTGCGTATCTTGTGGACGATGACGTTATGCAGCAGATAAAAGAGGGTGTCTTCCGTACTCGCGAAGAGATGGAAGAGTATCGCCATAGTCGCCTTCAAGAGGGCGCTAAGGTATACGCTGAGCAGTTCGGCATCGACCCTGAGGACGTTGATTATCAGCGTGGTTTCAACGGGGACATTACCGAGCGTAACATCTCGCTGTATGGTGCGCATGATAACTTCTTGAGCCGGCAAGCTCAGAAGGGCGCTATCATGAACAGCCGAGTGGAAc                                                                 >  1:466055/1‑301 (MQ=255)`  
                                                                                                                                                                                                                                           `gAAGCACTCCGAGTCAAGACTGGTCGTAACGCTGCGTATCTTGTGGACGATGACGTTATGCAGCAGATAAAAGAGGGTGTCTTCCGTACTCGCGAAGAGATGGAAGAGTATCGCCATAGTCGCCTTCAAGAGGGCGCTAAGGTATACGCTGAGCAGTTCGGCATCGACCCTGAGGACGTTGATTATCAGCGTGGTTTCAACGGGGACATTACCGAGCGTAACATCTCGCTGTATGGTGCGCATGATAACTTCTTGAGCCAGCAAGCTCAGAAGGGCGCTATCATGAACAGCCGAGTGGAAc                                                                 <  1:8187/301‑1 (MQ=255)`  
                                                                                                                                                                                                                                                      `aGTCAAGACTGGTCGTAACGCTGCGTATCTTGTGGACGATGACGTTATGCAGCAGATAAAAGAGGGTGTCTTCCGTACTCGCGAATAGATGGAAGAGTATCGCCATAGTCGCCTTCAAGAGGGCGCTAAGGTATACGCTGAGCAGTTCGGCATCGACCCTGAGGACGTTGATTATCAGCGTGGTTTCAACGGGGACATTACCGAGCGTAACATCTCGCTGTATGGTGCGCATGATAACTTCTTGAGCCAGCAAGCTCAGAAGGGCGCTATCATGAACAGCCGAGTGGAACTCCACGGTGTc                                                      >  1:652666/1‑301 (MQ=255)`  
                                                                                                                                                                                                                                                       `gTCAAGACTGGTCGTAACGCTGCGTATCTTGTGGACGATGACGTTATGCAGCAGATAAAAGAGGGTGTCTTCCGTACTCGCGAAGAGATGGAAGAGTATCGCCATAGTCGCCTTCAAGAGGGCGCTAAGGTATACGCTGAGCAGTTCGGCATCGACCATGAGGACGTTGATTATCAGCGTGGTTTCAACGGGGACATTACCGAGCGTAACATCTCTCTGTATGGTGCGCATGATAACTTCTTGAGCCAGCAAGCTCAGAAGGGCGCTATCATGAACAGCCgctgtctcttatacccatctc                                                     >  1:663097/1‑281 (MQ=255)`  
                                                                                                                                                                                                                                                          `aaGACTGGTCGTAACGCGGCGTATCTTGTGGACGATGACGTTATGCAGCAGATAAAAGAGGATGTCTTCCGTACTCGCGAAGAGATGGAAGAGTATCGCCATAGTCGCCTTCAAGAGGGCGCTAAGGTATACGCTGAGCAGTTCGGCATCGACCCTGAGGACGTTGATTATCGGCGTGGTTTCAACGGGGACATTACCGAGCGTAACATCTCGCTGTATGGTGCGCATGATAACTTCTTGAGCCAGCAAGCTCAGAAGGGCGCTATCATGAACAGCCGAGTGGAACTCAACGGTGTCCTTc                                                  <  1:364284/301‑1 (MQ=255)`  
                                                                                                                                                                                                                                                               `gggagaggtggataagagacaggtgGACGATGACGTTATGCAGCAGATAAAAGAGGGTGTCTTCCGTACTCGCGAAGAGATGGAAGAGTATCGCCATAGTCGCCTTCAAGAGGGCGCTAAGGTATACGCTGAGCAGTTCGGCATCGACCCTGAGGACGTTGATTATCAGCGTGGTTTCAACGGGGACATTACCGAGCGTAACATCTCGCTGTATGGTGCGCATGATAACTTCTTGAGCCAGCAAGCTCAGAAGGGCGCTATCATGAACAGCCGAGTGGAACTCAACGGTGTCCTTCAAGAc                                             <  1:75717/279‑1 (MQ=255)`  
                                                                                                                                                                                                                                                               `gggTCGTAACGTGGCGTATCGTGTGGACGATGACGTTATACAGCAGATAAAAGAGGGTGTCTGCCGTACTCGCGAAGAGATGGAAGAGTATCGCCATAGTCGCCTTCAAGAGGGCGCTAAGGTATAAGATGAGCAATTCGGCATCGACCCTGAGGACGTTGATTATCAGCTTGGTTTCAACGGGGACATTACCGAGCGTGACATCTAGCTGTATGGTGAGCAGGATAACTTCTTGAGCCAGCAAGCTCAGAAGGGCGCTATCATGAACAGCCGAGTGGAACTCAACGGTGTCCTTCAAGAc                                             <  1:416346/300‑1 (MQ=255)`  
                                                                                                                                                                                                                                                                `ggTCGTAACGCTGCGTATCTTGTGGACGATGACGTTATGCAGCAGATAAAAGAGGGTGTCTTCCGTACTCGCGAAGAGATGGAAGAGTATCGCCATAGTCGCCTTCAAGAGGGCGCTAAGGTATACGCTGAGCAGTTCGGCATCGACCCTGAGGACGTTGATTATCAGCGTGGTTTCAACGGGGACATTACCGAGCGTAACATCTCGCTGTATGGTGCGCATGATAACTTCTTGAGCCAGCAAGCTCAGAAGGGCGCTATCATGAACAGCCGAGTGGAACTCAACGGTGTCCTTCAAGAcc                                            >  1:232487/1‑301 (MQ=255)`  
                                                                                                                                                                                                                                                                 `ggcGTAACGCTGCGTATCTTGTGGACGATGACGTTATGCAGCACATAAAAGAGGGTGTCTTCCGTACTCGCGAAGAGATGGAAAAGTATCGCCATAGTCGCCGTCAAGAGGGCGCTAAGGTATACGCTGAGCAGTTCGGCATCGACCCTGAGGACGTTGATTATCAGCGTGGTTTCAACGGGGACATTACCGAGCGTAACATCTCGCTGTATGGTGCGCATGATAACTTCTTGAGCCAGCAAGCTCAGAAGGGCGCTATCATGAACAGCCGAGTGGAACTCAACGGTGTCCTTCAAGAccc                                           <  1:576630/299‑1 (MQ=255)`  
                                                                                                                                                                                                                                                                 `gTCGTAACGCTGCGTATCTTGTGGACGATGACGTTATGCAGCAGATACAAGAGGGTGTCTTCCGTACTTGCGAAGAGATGGAAGAGTATCGCCATAGTCCCCTTCAAGAGGGCGCTAAGGTATACGCTGAGCAGTTCGGCATCGACCCTGAGGACGTTGAATATCAGCGTGGTTTCAACGGGGCCATTACCGAGCGTAACATCTCGCTGTATGGTGCGCATGATAACTTCTTTAGCACGCAAGCTCAGAAGGGTGCTATCATGATCAGCCGAGTGGAACTCAACGGTGTCCtctaagctgt                                           >  1:634805/1‑292 (MQ=255)`  
                                                                                                                                                                                                                                                                     `taacttgtggTATCTTGGGGACGGTGAGTATATGCAGAGGATAAAAGATGGTGTCTTCCGTAATCGCGAAGAAATTGAAGAGTATTGCCATATTGGCCTTCAAGAGGGAGCTAAGGTATACGCTGAGCCGTTCGGCACCGACCCTGAGGACGTTGATTATCAGCGTGGTTTCAACGGGGACATTACCGAGCGTAACATCTCGCTATATGGTGCGCATGATGACTTCTTGAGCCAGCAAGCTCAGAAGGGCGCTATCATGAACAGCCGAGTGGAACTCAACGGTGTCCTTCAAGACCCTGat                                       <  1:538118/292‑1 (MQ=255)`  
                                                                                                                                                                                                                                                                       `aCGCTGCGTATCTTGTGGACGATGACGTTATGCAGCAGATAAAAGAGGGTGTCTTCCGTACTCGCGAAGAGATGGAAGAGTATCGCCATAGTCGCCTTCAAGAGGGCGCTAAGGTATACGCTGAGCAGTTCGGCATCGACCCTGAGGACGTTGATTATCAGCGGGGTTTCAACGGGGACATTACCGAGCGTAACATCTCGCTGTATGGTGCGCATGATAACTTCTTGAGCCAGCAAGCTCAGAAGGGCGCTATCATGAACAGCCGAGTGGAACTCAACGGTGTCCTTCAAGACCCTGatat                                     <  1:280893/301‑1 (MQ=255)`  
                                                                                                                                                                                                                                                                           `ggcggatcgagtgGACGATGACGTTAGGCAGAAGAAAAAAGAGGGTGTCATCCGTACTCGCGAAGAGATGGAAGAGTATCGCCATAGTCGCCTACAAGAGGGCGCTAAGGTATACGCTGAGCAGTGCGGCATCGACCCTGAGGACGTTGATTATCAGCGTGGTTTCAACGGGGACATTACCGAGCGTAACATCTCGCTGTAAGGTGCGCATGAAAACTTCTTGAGCCAGCAAGCTCAGAAGGGCGCTATCATGAACAGCCGAGTGGAACTCAACGGTGTCCTTCAAGACCCTGATAtgctg                                 <  1:143297/291‑1 (MQ=255)`  
                                                                                                                                                                                                                                                                                              `aaCGTGATGCAGCAGATAAAAGAGGGTTTCTTCCGTACTCGCGAAGAGATTGAAGAGTATCCCCTTATTAGCCTGAAAGCGGGCGCTAAGGTATTCGCTGAGCAGGTCGGCATCGACCCTGAGGACGTTGATTATCAGCGTGGTTTCAACGGGGACATTACCGAGCGTAACATCTCGCTGTATGGTGCGCATGATAACTTCTTGAGCCAGCAAGCTCAGAAGGGCGCTATCATGAACAGCCGAGTGGAACTCAACGGTGTCCTTCAAGACCCTGATATGCTGCGTCGTCCAGACTctgctg              <  1:329389/300‑1 (MQ=255)`  
                                                                                                                                                                                                                                                                                               `aCGTTATGCAGCAGATAAAAGAGGGTGTCTTCCGTACTCGCGAAGAGATGGAAGAGTATCGCCATAGTCGCCTTCAAGAGGGCGCTAAGGTATACGCTGAGCAGTTCGGCATCGACCCTGAGGACGTTGATTATCAGCGTGGTTTCAACGGGGACATTACCGAGCGTAACATCTCGCTGTATGGTGCGCATGATAACTTCTTGAGCCAGCAAGCTCAGAAGGGCGCTATCATGAACAGCCGAGTGGAACTCAACGGTGTCCTTCAAGACCCTGATATGCTGCGTCGTCCAGACTctgtctc             >  1:4711/1‑297 (MQ=255)`  
                                                                                                                                                                                                                                                                                                  `ttATGCAGCAGATAAAAGAGGGTGTCTTCCGTACTCGCGAAGAGATGGAAGAGTATCGCCATAGTCGCCTTCAAGAGGGCGCTAAGGTATACGCTGAGCAGTTCGGCATCGACCCTGAGGACGTTGATTATCAGCGTGGTTTCAACGGGGACATTACCGAGCGGAAAATCTGACTGTATGGTGCGAATGAAAACTTCTTGAGCCAGCAACCTCCGAAGGTCGCTATAAGGAACAGCCGATTGGAACTCAACGGTGTCCTTCAAGACCCGGATATGCTGCGCCGTCCAGACTCCGCTGActg          >  1:520131/1‑300 (MQ=255)`  
                                                                                                                                                                                                                                                                                                    `aTGCAGCAGATAAAAGAGGGTGTCTTCCGTACTCGCGAAGAGATGGAAGAGTATCGCCATAGTCGCCTTCAAGAGGGCGCTAAGGTATACGCTGAGCAGTTCGGCATCGACCCTGAGGACGTTGATTATCAGCGTGGTTTCAACGGGGACATTACCGAGCGTAACATCTCGCTGTATGGTGCGCATGATAACTTCTTGATCCAGCAAGCTCAGAAGGGCGCTATCATGAACAGCCGAGTGGAACTCAACGGTGTCCTTCAAGACCCTGATATGCTGCGTCGTCCAGACTCTGCTGActtct        >  1:155408/1‑301 (MQ=255)`  

                                                                                                                                                                                                                                                                                                          `|`                                                                                                                                                                                                                                                                                                              
`AGGTATGGGCTATCGTGCAGCAACCACTCAGGCCGAACAGCCAAGGTCAAGCCTATTGGACACCATTGGTCGGTTCGCTAAGGCTGGTGCCGATATGTATACCGCTAAGGAACAACGAGCACGAGACCTAGCTGATGAACGCTCTAACGAGATTATCCGTAAGCTGACCCCTGAGCAACGTCGAGAAGCTCTCAACAACGGGACCCTTCTGTATCAGGATGACCCATACGCTATGGAAGCACTCCGAGTCAAGACTGGTCGTAACGCTGCGTATCTTGTGGACGATGACGTTATGCAGAAGATAAAAGAGGGTGTCTTCCGTACTCGCGAAGAGATGGAAGAGTATCGCCATAGTCGCCTTCAAGAGGGCGCTAAGGTATACGCTGAGCAGTTCGGCATCGACCCTGAGGACGTTGATTATCAGCGTGGTTTCAACGGGGACATTACCGAGCGTAACATCTCGCTGTATGGTGCGCATGATAACTTCTTGAGCCAGCAAGCTCAGAAGGGCGCTATCATGAACAGCCGAGTGGAACTCAACGGTGTCCTTCAAGACCCTGATATGCTGCGTCGTCCAGACTCTGCTGACTTCTTTGAGA  >  NC_001604.1/28393‑28991`

  

Alignment Legend

Aligned base mismatch/match (shaded by quality score): `ATCG/ATCG` `< 3 ≤` `ATCG/ATCG` `< 9 ≤` `ATCG/ATCG` `< 19 ≤` `ATCG/ATCG` `< 34 ≤` `ATCG/ATCG` `< 38 ≤` `ATCG/ATCG`

Unaligned base: `atcg`    Masked matching base: `atcg`    Alignment gap: `‑`    Deleted base: `‑`
